# *Imago Mundi*, Imago AD, Imago ADNI

**DOI:** 10.1186/s13195-014-0062-5

**Published:** 2014-08-29

**Authors:** Victor L Villemagne, Seong Yoon Kim, Christopher C Rowe, Takeshi Iwatsubo

**Affiliations:** 1 Department of Nuclear Medicine and Centre for PET, Austin Health, 145 Studley Road, Heidelberg 3084, VIC, Australia; 2 The Florey Institute for Neurosciences and Mental Health, The University of Melbourne, 30 Royal Parade, Melbourne 3010, VIC, Australia; 3 Department of Medicine, The University of Melbourne, Grattan Street, Melbourne 3010, VIC, Australia; 4 Asan Medical Center, University of Ulsan Medical College, 88 Olympic-Ro 43-Gil, Songpa-Gu, Seoul, Korea; 5 Department of Neuropathology, School of Medicine, The University of Tokyo, 7-3-1, Hongo, Bunkyo-ku 113-0033, Tokyo, Japan

## Abstract

Since the launch in 2003 of the Alzheimer’s Disease Neuroimaging Initiative (ADNI) in the USA, ever growing, similarly oriented consortia have been organized and assembled around the world. The various accomplishments of ADNI have contributed substantially to a better understanding of the underlying physiopathology of aging and Alzheimer’s disease (AD). These accomplishments are basically predicated in the trinity of multimodality, standardization and sharing. This multimodality approach can now better identify those subjects with AD-specific traits that are more likely to present cognitive decline in the near future and that might represent the best candidates for smaller but more efficient therapeutic trials – trials that, through gained and shared knowledge, can be more focused on a specific target or a specific stage of the disease process. In summary, data generated from ADNI have helped elucidate some of the pathophysiological mechanisms underpinning aging and AD pathology, while contributing to the international effort in setting the groundwork for biomarker discovery and establishing standards for early diagnosis of AD.

## Introduction

Since the launch in 2003 of the Alzheimer’s Disease Neuroimaging Initiative (ADNI) in the USA, ever growing, similarly oriented consortia have been organized and assembled around the world. At the core of its conception, the ADNI is a multicenter and multidisciplinary approach for the study and longitudinal characterization of the natural history of aging and Alzheimer’s disease (AD) by means of structural and functional neuroimaging and fluid biomarkers along with clinical evaluations and neuropsychological assessments. Over the years, the aims and scope of the ADNI were expanded and refined, always aiming at increasing our understanding of the pathogenesis of AD – knowledge that can be now translated into more accurate and sensitive diagnostic and prognostic tests and techniques to guide therapeutic interventions by predicting and/or by monitoring response to therapy.

The ADNI proposed a novel and unique way of doing business. From the very beginning, data obtained by the study have been freely shared without embargo with the scientific community. For that purpose, data from the respective streams were centralized to ensure quality, and were made available to any registered researcher around the world. This sharing allows validation of results, as well as the identification of the most relevant and sensitive parameters at each stage of the disease that can be then translated into the design of therapeutic trials. This sharing also allows probing, challenging and comparing techniques for their diagnostic, prognostic and/or theragnostic value, testing new approaches, exploring new avenues and postulating new questions. This is perfectly illustrated by a case in which the same North American Alzheimer’s Disease Neuroimaging Initiative (NA-ADNI) magnetic resonance imaging (MRI) dataset yielded different longitudinal estimates of brain atrophy due to a methodological bias [1],[2], and how this bias was corrected [3]. While a series of steps were proposed to avoid similar issues in the future [4], this case underscored the need for a gold standard and highlighted how such a gold standard might need to be redefined under the new light of biomarkers [4],[5].

These issues have not discouraged researchers around the world, and they have been steadily and increasingly accessing and downloading ADNI data. The latest available reports show that ADNI data were accessed from more than 35 countries worldwide, by researchers at academic and governmental institutions, as well as by those in pharmaceutical and biotechnology industries [6],[7]. Furthermore, the ADNI in collaboration with the Alzheimer’s Association has put in motion international efforts for the standardization of biomarkers in order to increase compatibility between different centers.

**Figure 1:**
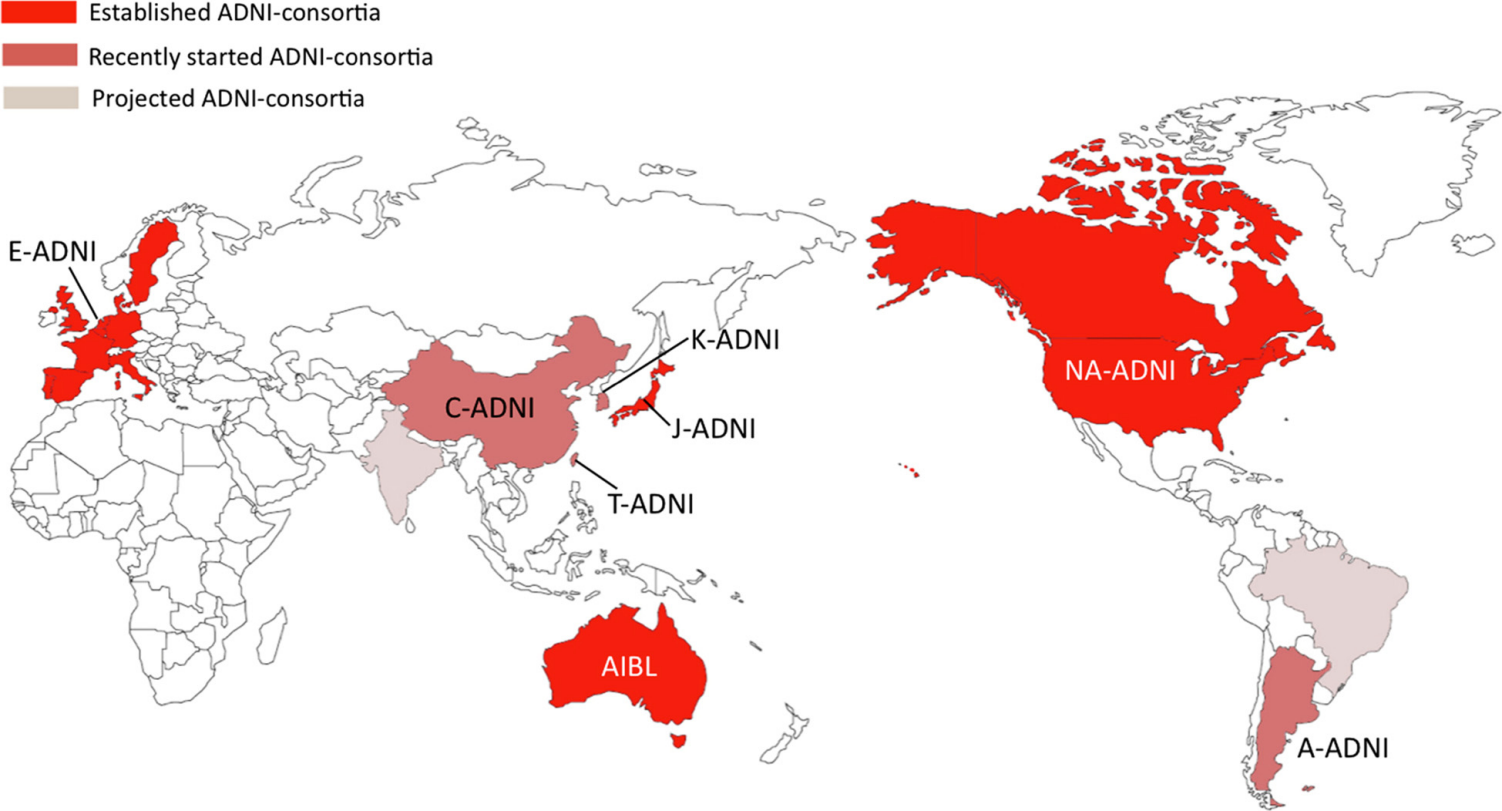
**World Wide Alzheimer’s Disease Neuroimaging Initiative*.*** Map of established (North America, Europe, Australia and Japan), recently started (Argentina, China, Korea and Taiwan), and projected (Brazil & India) Alzheimer’s Disease Neuroimaging Initiative (ADNI) sites. A-ADNI, Argentina Alzheimer’s Disease Neuroimaging Initiative; AIBL, Australian Imaging, Biomarkers and Lifestyle Flagship Study of Aging (Australian Alzheimer’s Disease Neuroimaging Initiative); C-ADNI, China Alzheimer’s Disease Neuroimaging Initiative; E-ADNI, European Alzheimer’s Disease Neuroimaging Initiative; J-ADNI, Japan Alzheimer’s Disease Neuroimaging Initiative; K-ADNI, Korea Alzheimer’s Disease Neuroimaging Initiative; NA-ADNI, North American Alzheimer’s Disease Neuroimaging Initiative; T-ADNI, Taiwan Alzheimer’s Disease Neuroimaging Initiative.

In response to the initial NA-ADNI, several similar initiatives for the study of AD were put in place in Australia (Australian Imaging, Biomarkers and Lifestyle Flagship Study of Aging (AIBL)), Japan (Japanese Alzheimer’s Disease Neuroimaging Initiative (J-ADNI)) and Europe (European Alzheimer’s Disease Neuroimaging Initiative (E-ADNI)). China, South Korea, Taiwan and Argentina have recently started recruiting participants, while other countries, such as Brazil and India, are at the planning stages of setting up like-minded consortia (Figure 1). This international approach will provide a multifaceted view of AD, finding the commonalities and differences across ethnicities and cultures, allowing cross-pollination and promotion of ideas, refinement of techniques, and improved assistance that can directly and/or indirectly affect the care, and potentially the cure, of this devastating disease. Furthermore, based on the ADNI model, imaging initiatives were organized around other neurodegenerative conditions such as the Parkinson Progression Marker Initiative [8].

We shall present an overview of each of these initiatives, trying to highlight their similarities and dissimilarities, accomplishments and pending issues. From the outset we know this is a Herculean task, and that much will be overlooked. The omissions will be more a reflection of the reviewers’ limitations than the initiatives they attempt to review.

## Established Alzheimer’s Disease Neuroimaging Initiative consortia

### North American Alzheimer’s Disease Neuroimaging Initiative

Since its inception in 2003, the NA-ADNI has focused on structural neuroimaging and fluid biomarkers for AD [9], assessing their validity as both surrogate markers for treatment trials and as a way to reduce trial cost and speed up drug development [10]. The ADNI was constituted as a public (National Institute on Aging, National Institute of Mental Health, National Institute of Biomedical Imaging and Bioengineering, National Institute for Alcohol Abuse and Alcoholism and the US Food and Drug Administration)–private (pharmaceutical companies along with private nonprofit philanthropic organizations, such as the Alzheimer’s Drug Discovery Foundation, Foundation of the National Institutes of Health and Alzheimer’s Association) collaboration, combining forces and sharing resources, infrastructure and information from diverse groups of academia, industry, nonprofit organizations and government in order to provide the foundation for the testing of new therapeutic approaches while at the same time speeding their development.

The initial phase of the NA-ADNI (ADNI 1) assessed more than 800 participants from about 60 centers in the USA and Canada. ADNI 1 was divided into five different cores: a neuroimaging core that included MRI [11] and fluorodeoxyglucose (FDG) positron emission tomography (PET); a clinical core that included the neuropsychological assessments; a biomarker core primarily focused on cerebrospinal fluid (CSF) and plasma analytes; a statistics core; and an informatics core. This initial phase included longitudinal assessments of cognition, MRI and blood in older cognitively unimpaired individuals, subjects classified as having mild cognitive impairment (MCI) and AD patients. About one-half of them also underwent lumbar puncture and/or FDG PET studies [12], and about 100 participants had amyloid-beta (Aβ) imaging with ^11^C-Pittsburgh Compound B (PiB) PET. To better characterize the prodromal stage of AD, a second phase of ADNI (ADNI-GO) incorporated individuals in the early stages of MCI [13]. The latest round of funding supported ADNI 2, which recruited ~700 new participants to the longitudinal study that, counting those participants retained from ADNI 1 and ADNI-GO, constitutes a cohort of about 1,100 participants.

Since its inception there has been a steady output of publications from the different cores of the NA-ADNI [7]. The tight collaboration of the different cores has already allowed integration of data, increasing the predictive power of the different markers along the spectrum of the disease but also to order them in time, with Aβ markers becoming abnormal first followed by changes in markers of neurodegeneration [14]. The clinical core, the cornerstone of all the other streams, has been refining and redefining the classifications of the different phases of the disease, trying to better characterize the early stages of the AD spectrum, evaluating not only their diagnostic performance but also their predictive power [15],[16]. The biomarkers core has focused on fluid biomarkers trying to better characterize the known AD profile in CSF [17],[18], but also searching for a plasma biomarker(s) profile that could predict disease progression especially at the predementia stages of the disease [14],[19]. Furthermore, longitudinal CSF data from NA-ADNI has made it possible to trace the dynamic changes in CSF Aβ, T-tau and p-tau over time, showing that Aβ becomes abnormal about 16 years before the onset of AD dementia [20]. The formation of the NA-ADNI and similar consortia allowed the combination of samples of multiple sites allowing new genome-wide association studies, which not only confirmed the relevance of certain gene candidates such as CLU and PICALM [21] but also revealed novel gene candidates and biological pathways [22].

The structural neuroimaging core has successfully standardized image acquisition and image processing and analysis protocols across multiple MRI scanners and across different MRI field strengths [11],[23], maintaining methodological consistency throughout the different stages of the NA-ADNI [24]. Protocols have been updated and all new enrollments undergo a 3T MRI and a core set of three sequences: a T1 magnetization-prepared rapid acquisition with gradient echo for brain volumetrics; fluid attenuated inversion recovery for the detection and quantification of white matter hyperintensities; and gradient echo MRI for the detection of microhemorrhages. Some centers also add new sequences such as arterial spin labeling for the quantification of brain perfusion, resting-state functional MRI to assess brain connectivity and diffusion tensor imaging for tractography mapping [24]. The PET core started by focusing on glucose metabolism and how to standardize studies acquired across multiple sites [12],[25],[26], standardization that has not only confirmed previous FDG findings [25],[27] but has also provided new insights through novel analytical paradigms [26],[28],[29]. The PET core was expanded to include Aβ imaging, initially with ^11^C-PiB [30] and then with ^18^F-florbetapir [31],[32]. More than 1,000 NA-ADNI participants have undergone ^18^F-florbetapir imaging.

The establishment of the NA-ADNI with its particular *weltanschauung* set in motion similar consortia around the world imbued of the same spirit, a movement that is gaining momentum and adherents; a movement that will provide an extraordinary wealth of data for the scientific world to share, dissect and examine for years to come.

### European Alzheimer’s Disease Neuroimaging Initiative

Founded in 2005, initially the E-ADNI was a pilot project funded by the Alzheimer's Association [33] in order to evaluate how many sites from a network of more than 50 clinical and research centers already participating in the European Alzheimer's Disease Consortium had the expertise, willingness and infrastructure to adopt the NA-ADNI structure and protocols [34]. The pilot study demonstrated not only that the selected centers were able to recruit healthy older volunteers as well as MCI and AD subjects, but that the clinical, imaging and biological data collected were equivalent to those from the NA-ADNI [34]. This pilot study led the European Commission to financially support the development of neuGRID in 2008 and neuroGRID4U later on [35].

One of the differences between the E-ADNI and the NA-ADNI was the inclusion of nonamnestic MCI as well as cases with subcortical vascular cognitive impairment and dementia. This approach allowed a wider assessment of different underlying etiologies of dementia, resulting in a more comprehensive look at aging.

The NA-ADNI and the E-ADNI had also joined forces in an effort to standardize and harmonize the landmarks and basic image analysis procedures for the segmentation of the hippocampus, measurement that can be used reliably and consistently across clinical settings and could eventually be useful as a surrogate marker for drug trials [36],[37]. A similar effort has concentrated on reaching a consensus for assessing FDG-PET data in a standardized unbiased way [38].

The collaboration between different European centers helped increase understanding of the clinical and predictive nature of Aβ imaging [39], and also helped to develop a predictive model combining information from MRI, FDG and CSF Aβ [40].

The efforts of the E-ADNI were not only concentrated in neuroimaging but also in fluid biomarkers, using the pilot data to validate and standardize results from across six different centers [41]. The integration of procedures and datasets of the ADNI with AddNeuroMed, a private–public consortium looking at the discovery of novel peripheral biomarkers, led to the identification and validation of markers such as α_2_-macroglobulin and complement factor C3 that are elevated in AD [42] and also of how plasma proteins correlated with markers of neurodegeneration [43] or cytokines combined with brain atrophy can be used as predictive markers of disease progression [44]. Further collaboration compared the diagnostic and predictive accuracy of several rating approaches for mesial temporal atrophy [45]-[47].

### Australian Alzheimer’s Disease Neuroimaging Initiative

The Australian ADNI, better known as the AIBL [48], was launched in 2006 as a prospective study of more than 1,000 older individuals [49],[50]. One of the distinctive features of AIBL was the inclusion of a large proportion (~70%) of healthy older individuals who, along with MCI (12%) and AD subjects (20%), were followed-up every 18 months, collecting data on demographic, genetic and lifestyle factors, cognitive function and fluid biomarkers. About one-third of the participants underwent structural MRI and Aβ imaging scans with PiB PET [50],[51]. While the AIBL adopted a very similar approach to neuropsychological assessments, blood biomarkers and structural MRI, the approach to disease-specific biomarkers differed – the AIBL concentrated from the very beginning on Aβ imaging, while the NA-ADNI initially focused on FDG and CSF biomarkers.

Another particular feature of the AIBL is the inclusion of a dedicated lifestyle research stream assessing the effects of exercise and/or dietary modifications on cognition or biomarkers, which showed that higher intensity exercise was associated with better cognitive function in older controls [52]. In the area of fluid biomarkers, lower plasma Aβ_1–42_/Aβ_1–40_ ratio was associated with disease progression in controls and with brain Aβ in MCI and AD cases [53]. Also, AD patients had significantly lower total plasma apolipoprotein E (ApoE) levels than controls or MCI patients [54], but higher luteinizing hormone in serum [55]. A perfect example of the cross-validation afforded by sharing databases is an AIBL study where a panel of biomarkers predicting brain Aβ burden with a sensitivity and specificity of 80% and 82%, respectively, were further validated using independent biomarker data from the NA-ADNI [56].

Initial neuroimaging results showed that the prevalence of high Aβ burden (Aβ+) in cognitively unimpaired individuals increased with age, and was higher in individuals carrying at least one APOE ε4 allele [51]. Furthermore, the Aβ burden was related to worse cognitive performance in cognitively unimpaired females, raising questions with regard to gender susceptibility to Aβ [57]. While memory in the cognitively unimpaired adults with low Aβ burden (Aβ–) remained stable over 18 months, all aspects of episodic memory were observed to deteriorate substantially in Aβ + nondemented participants [58],[59].

From a clinical perspective, some biomarkers have been shown to serve as predictors of disease progression. For example, Aβ imaging data demonstrated that Aβ + amnestic MCI cases were much more likely to progress to AD over 18 to 36 months than Aβ– MCI cases [60],[61]. Subtle memory impairment in Aβ + healthy individuals indicated that these individuals were at high risk to progress to MCI or AD within 3 years [61]. Aβ deposition was also found to be strongly related to grey matter atrophy, where the rates of grey matter atrophy were significantly higher in Aβ + cognitively unimpaired individuals [62],[63]. Moreover, the hippocampal volume and temporal Aβ deposition provided independent contributions to memory deficits, suggesting that both factors should be independently targeted in therapeutic trials aimed at reducing cognitive decline [64]. These associations were not observed at the MCI and AD stages, suggesting that other pathological, probably downstream, events might be responsible for the progressive atrophy and cognitive decline [65]. When assessing whether brain-derived neurotrophic factor polymorphism moderated the relationship between cognition, brain volume and Aβ burden in cognitively unimpaired individuals, carrying a Met allele was associated with moderate decline in episodic memory, reductions in hippocampal volume and increased risk of progression to MCI when compared with Val/Val homozygotes [66], but that it did not affect rates of Aβ accumulation [66].

The prospective longitudinal design of the study allowed the examination of changes in Aβ burden over time, where small but significant increases in neocortical Aβ burden were observed in the AD and MCI groups, and in Aβ + controls, confirming the notion that Aβ deposition precedes cognitive impairment [60]. Furthermore, higher rates of Aβ deposition were associated with higher Aβ burden and identified the existence of Aβ accumulators and Aβ nonaccumulators, with Aβ accumulators even found among Aβ– controls [67]. Consequently, Aβ imaging data from the 3-year follow-up were then used to calculate the rates of Aβ deposition over time, showing that Aβ deposition is a slow and protracted process that takes about two decades to go from the threshold of abnormal Aβ burden to the levels usually observed in AD [68] and that Aβ deposition precedes by more than a decade hippocampal atrophy and memory impairment [68].

Renovated efforts are aimed at obtaining structural and Aβ imaging on all participants enrolled in the study. To date, the AIBL has enrolled almost an additional 600 individuals undergoing longitudinal Aβ imaging studies either with ^11^C-PiB (*n* = 65), ^18^F-flutemetamol (*n* = 204), ^18^F-florbetaben (*n* = 127) or ^18^F-florbetapir (*n* = 195).

### Japanese Alzheimer’s Disease Neuroimaging Initiative

Discussions for the launch of the J-ADNI [69] started in 2006, as a result of the urgent need to meet the requirements for global clinical trials of AD disease-modifying drugs that were about to start in Japan. Japanese neurologists, psychiatrists, geriatricians and neuroradiologists, despite their high scientific performance in dementia, had little experience in nationwide or global-level clinical studies of AD [70],[71]. Second, Japan did not have sufficient infrastructures, such as the Alzheimer’s Disease Cooperative Study, that are required to conduct clinical studies or trials of AD. Therefore, in 2007 the J-ADNI was funded by the two major governmental funding agencies: the Ministry of Health, Labor and Welfare; and the New Energy and Industrial Technology Development Organization (a foundation of the Ministry of Economy, Technology, and Industry) [70],[71]. Seven domestic pharmas (Astellas, Eisai, Daiichi-Sankyo, Dainippon-Sumitomo, Shionogi, Takeda, Tanabe-Mitsubishi) and four international pharmas (Bristol-Myers Squibb, Eli-Lilly, Merck-Banyu, Pfizer) organized an industry scientific advisory board and contributed one-third of the total 500,000,000¥/year J-ADNI budget.

The J-ADNI research protocol was designed to maximize compatibility with the NA-ADNI, including structural MRI, FDG and Aβ imaging with PET, CSF sampling and APOE genotyping, combined with a set of clinical and psychometric tests. The initially planned sample size for the study was 300 individuals with amnestic MCI, 150 early AD individuals and 150 cognitively normal individuals. In total, 38 clinical sites participated in the J-ADNI. The clinical core ensures that the registration and clinical evaluation of the participants closely collaborates with the neuropsychology core, the latter being responsible for maximizing the harmonization between English and Japanese versions of psychometric tests. The MRI core has established an algorithm to achieve the standardization of MRI scans among clinical sites using different MRI scanners from various vendors, based on a three-dimensional magnetization-prepared rapid acquisition with gradient echo scan protocol using the ADNI phantom. Researchers at the J-ADNI have created programs for the correction and calibration of signal equity or distortion of the images, which enabled the rigorous volumetric analysis of MRI data [72]. The PET core has established the standardized protocol for PET imaging in the J-ADNI. Twenty-eight sites are conducting FDG PET, covering ~67% of participants, and analysis of the multicenter data has started [73]-[75]. Aβ imaging using ^11^C-PiB started in ~15 sites, with two sites also performing Aβ imaging studies using ^11^C-BF-227. About 40% of the J-ADNI cohort had undergone Aβ imaging. The biomarker core established the J-ADNI biosample repository in Niigata University, centralizing the nationwide collection network of biofluid samples. Blood samples are collected from all participants at every visit, and ~40% of the participants had lumbar puncture for CSF samples without severe adverse events. The biomarker core has recently achieved the harmonization of protocol for X-MAP quantitation of CSF samples using Alzbio3 enzyme-linked immunosorbent assay, in collaboration with NA-ADNI biomarker core and Innogenetics. The APOE genotype is also characterized at the Niigata site.

By spring 2012 the 38 clinical sites had enrolled 545 participants meeting the inclusion criteria (239 amnestic MCI individuals, 154 cognitively normal older individuals, and 152 early AD individuals). More than 3,600 visits (that comprise ~97% of the total scheduled visits) were completed by autumn 2013, and all data continue to be analyzed, with analysis to be completed by mid 2014. Briefly, the conversion rate of late amnestic MCI individuals appears to be slightly higher than those in the NA-ADNI, at similar levels to those of the late amnestic MCI cases from the National Alzheimer’s Coordinating Center, USA, with Aβ-positive MCI individuals exhibiting higher rates of conversion to dementia. The rates of hippocampal atrophy were comparable with those measured in the NA-ADNI and, as in the NA-ADNI, lower Aβ_1–42_ in CSF was in good accordance with Aβ + PET scans.

Currently, the second phase of the J-ADNI (J-ADNI2) is being launched, focusing on the two populations of older individuals with very early stages of AD; that is, preclinical AD and early/late amnestic MCI. The first participant was screened on 11 December 2013. J-ADNI2 is comprised of two studies. The first is aimed at preclinical AD, where ~700 cognitively normal older individuals will undergo Aβ imaging at 31 PET sites with one of several probes, either ^11^C-PiB, ^18^F-florbetapir or ^18^F-flutemetamol. The scans will allow selection and recruitment of 150 cognitively normal, Aβ + individuals meeting the criteria of preclinical AD that will be followed-up in an annual fashion for 3 years. The second study of J-ADNI2 is the MCI study, devoted to the longitudinal investigation of early and late amnestic MCI, according to the criteria used by the NA-ADNI2. CSF sampling and Aβ imaging, together with structural MRI using 3T scanners and either of the three optional sequences (resting-state functional MRI, arterial spin labeling, or diffusion tensor imaging) will be performed in all participants of preclinical AD and MCI studies. These studies will pave the way towards the upcoming clinical trials of prodromal AD (or MCI due to AD) and the future very early treatment of preclinical AD in Japan, similar to the A4 anti-Aβ treatment of asymptomatic AD in the USA.

## More recent Alzheimer’s Disease Neuroimaging Initiative consortia

Argentina, China, Korea and Taiwan have recently joined the World Wide Alzheimer’s Disease Neuroimaging Initiative (WW-ADNI)-like efforts in the study of AD. Another two countries are establishing the platforms to launch their respective studies. With arrangements already in place, Brazil plans to join the WW-ADNI in 2014 – aiming at the study of healthy older controls, individuals reporting subjective memory complaints, MCI and AD patients – while India is trying to secure funding for their planned 5-year prospective study.

### Argentina Alzheimer’s Disease Neuroimaging Initiative

After institutional review board approval in 2011, the Argentina Alzheimer’s Disease Neuroimaging Initiative has already recruited 60 participants, with plans to expand the cohort to 300 participants. To date, most participants have undergone CSF and blood examination, MRI, FDG and PiB scans. A unique characteristic of the Argentina Alzheimer’s Disease Neuroimaging Initiative is the intention to use the same methodological approach to study the adult offspring of AD patients that present with autonomic and circadian abnormalities. The results from the initial CSF assessments were presented in Boston in 2013 [76].

### China Alzheimer’s Disease Neuroimaging Initiative

The China Alzheimer’s Disease Neuroimaging Initiative is planned as a 5-year prospective study aiming at recruiting 800 to 1,000 participants in the first 2 years, with an emphasis on MCI subjects. Clinical and neuropsychological evaluation will be performed every 6 months, with fluid biomarkers and neuroimaging every year. After fluid biomarker, MRI and FDG protocols were established and implemented, recruitment started in 2012. Aβ imaging with ^18^F-florbetapir is planned to commence in 2014. At the last WW-ADNI update in Boston in 2013, 16 subjects had already been enrolled, with 11 of them having completed a 1-year follow-up [77].

### Korea Alzheimer’s Disease Neuroimaging Initiative

The Korea Alzheimer’s Disease Neuroimaging Initiative is a project funded by both industry and the Korean Ministry of Health and Welfare. This 6-year project aims at recruiting 500 participants for the initial 2 years, and then following them up annually. A distinctive and important feature of the Korea Alzheimer’s Disease Neuroimaging Initiative is that it will enroll in the study, in a more vigorous way than the E-ADNI, individuals with either vascular MCI or subcortical vascular dementia. Twenty-five nationwide clinical sites will recruit and monitor the progress of 50 healthy controls, 200 MCI individuals, 100 vascular MCI individuals, 50 AD individuals and 100 subcortical vascular dementia subjects. As with the other ADNI initiatives, participants will be characterized and followed-up with cognitive tests, fluid (including blood, CSF and ApoE genotype) and neuroimaging (MRI, FDG and Aβ imaging with ^18^F-flutemetamol) biomarkers. Given the relatively large proportion of Asian patients affected by vascular dementia and continuing an already established line of investigation [78], the Korea Alzheimer’s Disease Neuroimaging Initiative plans to evaluate the effects of vascular risk factors on the development and progression of AD and subcortical vascular dementia. At the WW-ADNI meeting in Boston in 2013 [77], the Korea Alzheimer’s Disease Neuroimaging Initiative reported to be in the final stages of setting up the infrastructure for the study and ready to start recruiting early in 2014.

### Taiwan Alzheimer’s Disease Neuroimaging Initiative

The Taiwan Alzheimer’s Disease Neuroimaging Initiative plans to enroll 200 older individuals following the same criteria as ADNI-GO that, besides enrolling a group of AD patients and a group of age-matched healthy controls, separates the MCI subjects into early or late MCI. The initial phase of the study will involve six centers, eventually expanding to a total of 12 centers across Taiwan. Participants will undergo cognitive tests and testing for fluid biomarkers. Neuroimaging will comprise extensive three-dimensional MRI (volumetry, diffusion tensor imaging, resting-state functional MRI, susceptibility weighted imaging and magnetic resonance spectroscopy) as well as FDG and building on their previous Aβ imaging experience with ^18^F-florbetapir [79]-[81].

## Limitations

The limitations of the ADNI model are often acknowledged by ADNI researchers [7]. Some of these limitations are related to the study design, some of them are related to data collection, storage and analysis, and some are related to the potential misuse of the shared data. The study was designed as an observational, natural progression study, not as a clinical trial, so while it might identify relevant biomarkers and establish their behavior over time, it cannot predict how or how much those biomarkers will change in response to therapy. Another limitation is that the study uses sharply defined clinical groups in a specific age range, with a particular emphasis on memory impairment that might preclude the generalization of the findings to the general population; therefore, the study is not ideal to test the diagnostic properties of some biomarkers. Other studies, evaluating a large sample of individuals from the general population with a wide variety of comorbidities, will be required to assess the real impact of the contributions of the ADNI.

Another set of limitations are related to data collection, including how and when biological samples are collected, stored and analyzed [82], image quality across time and across centers, as well as what is not being collected or what other techniques are not being used [7]. For example, not all tests are administered to all participants. Within the same cohort this is illustrated by the fact that not all NA-ADNI participants undergoing CSF examination had FDG scans, and across cohorts by the fact that, while participants from the NA-ADNI had both FDG and CSF collected, these tests were not performed in the AIBL cohort. Another issue is missing data due to attrition (for example, death, dropout, or withdrawal) as well as some data that had to be discarded due to poor quality.

Finally, another issue that has been raised against ADNI results is that these results are mainly confirmatory. While the argument is valid in some cases, what is overlooked is that the confirmation, as well as the refutation, of the usefulness of certain biomarkers was performed in a large cohort of individuals, in contrast with the smaller samples used in the original evaluations. Not only that, the longitudinal design of the study also allows for the assessment of the change of those biomarkers over time. This cross-sectional and longitudinal validation of biomarkers in a large cohort is a necessary step in order to translate these biomarkers into clinical practice.

## Conclusions

The prospective and multidisciplinary approach adopted by the ADNI has made substantial contributions to the understanding of cognitive and physiological changes associated with aging in general, and the AD spectrum in particular. Furthermore, the identification and validation in a large cohort of specific biomarkers provides a framework to assess AD risk in individuals prior to the onset of the clinical phenotype, while eventually allowing evaluation of the therapeutic and lifestyle interventions likely to emerge within the next decade aimed at preventing or delaying the onset of symptoms. Data gathered from different ADNI sites concurred in showing that some of the pathological changes characteristic of the disease are already present at the preclinical and prodromal stages, eventually leading to the postulation of new diagnostic criteria based on markers of pathology (Aβ) and markers of neuronal injury [83]-[85]. The implementation of the new staging criteria for preclinical AD [84] in an independent cohort showed that about 25% of the cases presented with AD-like neurodegeneration but no significant Aβ deposition, leading to the introduction of an additional category labeled ‘suspected non-Alzheimer’s disease pathology’ [86]. Some of these biomarkers have also been used to predict the rate of evolution of the preclinical changes and the onset of the clinical phase of AD, crucial for the design and timing of disease-specific interventions. For example, CSF [20],[68] and Aβ imaging studies [20],[68] have shown that extensive Aβ deposition precedes significant cognitive impairment by more than 15 to 20 years, results that were further validated in a different cohort [87]. One issue to be highlighted is that these estimates of a process that probably extends decades were derived from still relatively short longitudinal assessments. Longer follow-ups will be required to reassess and revise the current results [20],[68],[87].

We are still not aware of the full impact of the ADNI. Only time will be able to judge the enormous contribution, founding blueprint, and insightful roadmap that the ADNI provided to the study of AD. What we do know is that the ADNI way of doing business, sharing data and joining efforts, setting standards and fostering collaborations, multimodal approaches and international cooperation are already among its most remarkable accomplishments, and that these interactions and cross-pollination inform, reform and transform ongoing and newly developed research protocols, while at the same time setting standards and validating biomarkers and techniques that can hopefully be translated to clinical practice and disease-modifying therapeutic trials. We are also acutely aware that we aimed at describing the ocean, and as yet have only been able to provide a glass of water – such is the complexity of the study of AD.

## Abbreviations

AD: Alzheimer’s disease

ADNI: Alzheimer’s Disease Neuroimaging Initiative

AIBL: Australian Imaging, Biomarkers and Lifestyle Flagship Study of Aging

ApoE: Apolipoprotein E

Aβ: Amyloid-beta

Aβ–: Low amyloid burden

Aβ+: High amyloid burden

CSF: Cerebrospinal fluid

E-ADNI: European Alzheimer’s Disease Neuroimaging Initiative

FDG: Fluorodeoxyglucose

J-ADNI: Japanese Alzheimer’s Disease Neuroimaging Initiative

MCI: Mild cognitive impairment

MRI: Magnetic resonance imaging

NA-ADNI: North American Alzheimer’s Disease Neuroimaging Initiative

PET: Positron emission tomography

PiB: Pittsburgh Compound B

WW-ADNI: World Wide Alzheimer’s Disease Neuroimaging Initiative

## Competing interests

As leaders for the respective ADNI consortia, SYK, CRC and TI received funds from governmental institutions and private pharmaceutical companies along with private nonprofit philanthropic organizations. The funding sources had no input in the design or writing of the review. VLV declares that he has no competing interests.

## Authors’ contributions

VLV and TI participated in the design and writing of this review, and drafting of the manuscript. All authors were responsible for critical revision of the manuscript for important intellectual content. All authors read and approved the final manuscript.

## References

[B1] Hua X Lee S Hibar DP Yanovsky I Leow AD Toga AW Jack CR Jr Bernstein MA Reiman EM Harvey DJ Kornak J Schuff N Alexander GE Weiner MW Thompson PM Mapping Alzheimer’s disease progression in 1309 MRI scans: power estimates for different inter-scan intervals Neuroimage 2010 51 63 75 20139010 10.1016/j.neuroimage.2010.01.104 PMC2846999

[B2] Thompson WK Holland D Bias in tensor based morphometry Stat-ROI measures may result in unrealistic power estimates Neuroimage 2011 57 1 4 21349340 10.1016/j.neuroimage.2010.11.092 PMC3471806

[B3] Hua X Gutman B Boyle CP Rajagopalan P Leow AD Yanovsky I Kumar AR Toga AW Jack CR Jr Schuff N Alexander GE Chen K Reiman EM Weiner MW Thompson PM Accurate measurement of brain changes in longitudinal MRI scans using tensor-based morphometry Neuroimage 2011 57 5 14 21320612 10.1016/j.neuroimage.2011.01.079 PMC3394184

[B4] Fox NC Ridgway GR Schott JM Algorithms, atrophy and Alzheimer’s disease: cautionary tales for clinical trials Neuroimage 2011 57 15 18 21296168 10.1016/j.neuroimage.2011.01.077

[B5] Scheltens P Rockwood K How golden is the gold standard of neuropathology in dementia? Alzheimers Dement 2011 7 486 489 21784357 10.1016/j.jalz.2011.04.011

[B6] Toga AW Crawford KL The informatics core of the Alzheimer’s Disease Neuroimaging Initiative Alzheimers Dement 2010 6 247 256 20451873 10.1016/j.jalz.2010.03.001 PMC2927123

[B7] Weiner MW Veitch DP Aisen PS Beckett LA Cairns NJ Green RC Harvey D Jack CR Jagust W Liu E Morris JC Petersen RC Saykin AJ Schmidt ME Shaw L Siuciak JA Soares H Toga AW Trojanowski JQ The Alzheimer’s Disease Neuroimaging Initiative: a review of papers published since its inception Alzheimers Dement 2012 8 S1 S68 22047634 10.1016/j.jalz.2011.09.172 PMC3329969

[B8] **The Parkinson Progression Marker Initiative (PPMI).** *Prog Neurobiol* 2011, **95:**629–635. 10.1016/j.pneurobio.2011.09.005 PMC9014725 21930184

[B9] http://www.adni-info.org/Home.aspx *North American Alzheimer’s Disease Neuroimaging Initiative* []

[B10] Mueller SG Weiner MW Thal LJ Petersen RC Jack CR Jagust W Trojanowski JQ Toga AW Beckett L Ways toward an early diagnosis in Alzheimer’s disease: the Alzheimer’s Disease Neuroimaging Initiative (ADNI) Alzheimers Dement 2005 1 55 66 17476317 10.1016/j.jalz.2005.06.003 PMC1864941

[B11] Jack CR Jr Bernstein MA Fox NC Thompson P Alexander G Harvey D Borowski B Britson PJ Whitwell L Jr Ward C Dale AM Felmlee JP Gunter JL Hill DL Killiany R Schuff N Fox-Bosetti S Lin C Studholme C DeCarli CS Krueger G Ward HA Metzger GJ Scott KT Mallozzi R Blezek D Levy J Debbins JP Fleisher AS Albert M The Alzheimer’s Disease Neuroimaging Initiative (ADNI): MRI methods J Magn Reson Imaging 2008 27 685 691 18302232 10.1002/jmri.21049 PMC2544629

[B12] Jagust WJ Bandy D Chen K Foster NL Landau SM Mathis CA Price JC Reiman EM Skovronsky D Koeppe RA The Alzheimer’s Disease Neuroimaging Initiative positron emission tomography core Alzheimers Dement 2010 6 221 229 20451870 10.1016/j.jalz.2010.03.003 PMC2920531

[B13] Weiner MW Aisen PS Jack CR Jr Jagust WJ Trojanowski JQ Shaw L Saykin AJ Morris JC Cairns N Beckett LA Toga A Green R Walter S Soares H Snyder P Siemers E Potter W Cole PE Schmidt M The Alzheimer’s disease neuroimaging initiative: progress report and future plans Alzheimers Dement 2010 6 202 211 e7 20451868 10.1016/j.jalz.2010.03.007 PMC2927112

[B14] Trojanowski JQ Vandeerstichele H Korecka M Clark CM Aisen PS Petersen RC Blennow K Soares H Simon A Lewczuk P Dean R Siemers E Potter WZ Weiner MW Jack CR Jr Jagust W Toga AW Lee VM Shaw LM Update on the biomarker core of the Alzheimer’s Disease Neuroimaging Initiative subjects Alzheimers Dement 2010 6 230 238 20451871 10.1016/j.jalz.2010.03.008 PMC2867838

[B15] Petersen RC Aisen PS Beckett LA Donohue MC Gamst AC Harvey DJ Jack CR Jr Jagust WJ Shaw LM Toga AW Trojanowski JQ Weiner MW Alzheimer’s Disease Neuroimaging Initiative (ADNI): clinical characterization Neurology 2010 74 201 209 20042704 10.1212/WNL.0b013e3181cb3e25 PMC2809036

[B16] Aisen PS Petersen RC Donohue MC Gamst A Raman R Thomas RG Walter S Trojanowski JQ Shaw LM Beckett LA Jack CR Jr Jagust W Toga AW Saykin AJ Morris JC Green RC Weiner MW Clinical Core of the Alzheimer’s Disease Neuroimaging Initiative: progress and plans Alzheimers Dement 2010 6 239 246 20451872 10.1016/j.jalz.2010.03.006 PMC2867843

[B17] Shaw LM Vanderstichele H Knapik-Czajka M Clark CM Aisen PS Petersen RC Blennow K Soares H Simon A Lewczuk P Dean R Siemers E Potter W Lee VM Trojanowski JQ Cerebrospinal fluid biomarker signature in Alzheimer’s disease neuroimaging initiative subjects Ann Neurol 2009 65 403 413 19296504 10.1002/ana.21610 PMC2696350

[B18] Shaw LM Vanderstichele H Knapik-Czajka M Figurski M Coart E Blennow K Soares H Simon AJ Lewczuk P Dean RA Siemers E Potter W Lee VM Trojanowski JQ Qualification of the analytical and clinical performance of CSF biomarker analyses in ADNI Acta Neuropathol 2011 121 597 609 21311900 10.1007/s00401-011-0808-0 PMC3175107

[B19] Toledo JB Korff A Shaw LM Trojanowski JQ Zhang J CSF alpha-synuclein improves diagnostic and prognostic performance of CSF tau and Abeta in Alzheimer’s disease Acta Neuropathol 2013 126 683 697 23812319 10.1007/s00401-013-1148-z PMC3812407

[B20] Toledo JB Xie SX Trojanowski JQ Shaw LM Longitudinal change in CSF Tau and Abeta biomarkers for up to 48 months in ADNI Acta Neuropathol 2013 126 659 670 23812320 10.1007/s00401-013-1151-4 PMC3875373

[B21] Jun G Naj AC Beecham GW Wang LS Buros J Gallins PJ Buxbaum JD Ertekin-Taner N Fallin MD Friedland R Inzelberg R Kramer P Rogaeva E St George-Hyslop P Cantwell LB Dombroski BA Saykin AJ Reiman EM Bennett DA Morris JC Lunetta KL Martin ER Montine TJ Goate AM Blacker D Tsuang DW Beekly D Cupples LA Hakonarson H Meta-analysis confirms CR1, CLU, and PICALM as Alzheimer disease risk loci and reveals interactions with APOE genotypes Arch Neurol 2010 67 1473 1484 20697030 10.1001/archneurol.2010.201 PMC3048805

[B22] Ramanan VK Kim S Holohan K Shen L Nho K Risacher SL Foroud TM Mukherjee S Crane PK Aisen PS Petersen RC Weiner MW Saykin AJ Genome-wide pathway analysis of memory impairment in the Alzheimer’s Disease Neuroimaging Initiative (ADNI) cohort implicates gene candidates, canonical pathways, and networks Brain Imaging Behav 2012 6 634 648 22865056 10.1007/s11682-012-9196-x PMC3713637

[B23] Wyman BT Harvey DJ Crawford K Bernstein MA Carmichael O Cole PE Crane PK DeCarli C Fox NC Gunter JL Hill D Killiany RJ Pachai C Schwarz AJ Schuff N Senjem ML Suhy J Thompson PM Weiner M Jack CR Jr Standardization of analysis sets for reporting results from ADNI MRI data Alzheimers Dement 2013 9 332 337 23110865 10.1016/j.jalz.2012.06.004 PMC3891834

[B24] Jack CR Jr Bernstein MA Borowski BJ Gunter JL Fox NC Thompson PM Schuff N Krueger G Killiany RJ Decarli CS Dale AM Carmichael OW Tosun D Weiner MW Update on the magnetic resonance imaging core of the Alzheimer’s disease neuroimaging initiative Alzheimers Dement 2010 6 212 220 20451869 10.1016/j.jalz.2010.03.004 PMC2886577

[B25] Langbaum JB Chen K Lee W Reschke C Bandy D Fleisher AS Alexander GE Foster NL Weiner MW Koeppe RA Jagust WJ Reiman EM Categorical and correlational analyses of baseline fluorodeoxyglucose positron emission tomography images from the Alzheimer’s Disease Neuroimaging Initiative (ADNI) Neuroimage 2009 45 1107 1116 19349228 10.1016/j.neuroimage.2008.12.072 PMC2886795

[B26] Haense C Herholz K Jagust WJ Heiss WD Performance of FDG PET for detection of Alzheimer’s disease in two independent multicentre samples (NEST-DD and ADNI) Dement Geriatr Cogn Disord 2009 28 259 266 19786778 10.1159/000241879 PMC7077083

[B27] Habeck C, Risacher S, Lee GJ, Glymour MM, Mormino E, Mukherjee S, Kim S, Nho K, DeCarli C, Saykin AJ, Crane PK: **Relationship between baseline brain metabolism measured using [**^**18**^**F]FDG PET and memory and executive function in prodromal and early Alzheimer’s disease.** *Brain Imaging Behav* 2012, **6:**568–583. 10.1007/s11682-012-9208-x PMC3532575 23179062

[B28] Chen K Langbaum JB Fleisher AS Ayutyanont N Reschke C Lee W Liu X Bandy D Alexander GE Thompson PM Foster NL Harvey DJ de Leon MJ Koeppe RA Jagust WJ Weiner MW Reiman EM Twelve-month metabolic declines in probable Alzheimer’s disease and amnestic mild cognitive impairment assessed using an empirically pre-defined statistical region-of-interest: findings from the Alzheimer’s Disease Neuroimaging Initiative Neuroimage 2010 51 654 664 20202480 10.1016/j.neuroimage.2010.02.064 PMC2856742

[B29] Landau SM Harvey D Madison CM Koeppe RA Reiman EM Foster NL Weiner MW Jagust WJ Associations between cognitive, functional, and FDG-PET measures of decline in AD and MCI Neurobiol Aging 2011 32 1207 1218 19660834 10.1016/j.neurobiolaging.2009.07.002 PMC2891865

[B30] Apostolova LG Hwang KS Andrawis JP Green AE Babakchanian S Morra JH Cummings JL Toga AW Trojanowski JQ Shaw LM Jack CR Jr Petersen RC Aisen PS Jagust WJ Koeppe RA Mathis CA Weiner MW Thompson PM 3D PIB and CSF biomarker associations with hippocampal atrophy in ADNI subjects Neurobiol Aging 2010 31 1284 1303 20538372 10.1016/j.neurobiolaging.2010.05.003 PMC3051831

[B31] Landau SM Breault C Joshi AD Pontecorvo M Mathis CA Jagust WJ Mintun MA Amyloid-beta imaging with Pittsburgh compound B and florbetapir: comparing radiotracers and quantification methods J Nucl Med 2013 54 70 77 23166389 10.2967/jnumed.112.109009 PMC3747730

[B32] Landau SM Mintun MA Joshi AD Koeppe RA Petersen RC Aisen PS Weiner MW Jagust WJ Amyloid deposition, hypometabolism, and longitudinal cognitive decline Ann Neurol 2012 72 578 586 23109153 10.1002/ana.23650 PMC3786871

[B33] http://www.centroalzheimer.it/E-ADNI_project.htm *European Alzheimer’s Disease Neuroimaging Initiative* []

[B34] Frisoni GB Henneman WJ Weiner MW Scheltens P Vellas B Reynish E Hudecova J Hampel H Burger K Blennow K Waldemar G Johannsen P Wahlund LO Zito G Rossini PM Winblad B Barkhof F The pilot European Alzheimer’s Disease Neuroimaging Initiative of the European Alzheimer’s Disease Consortium Alzheimers Dement 2008 4 255 264 18631976 10.1016/j.jalz.2008.04.009 PMC2657833

[B35] Frisoni GB Alzheimer’s disease neuroimaging initiative in Europe Alzheimers Dement 2010 6 280 285 20451877 10.1016/j.jalz.2010.03.005

[B36] Boccardi M Bocchetta M Ganzola R Robitaille N Redolfi A Duchesne S Jack CR Jr Frisoni GB Operationalizing protocol differences for EADC-ADNI manual hippocampal segmentation Alzheimers Dement 2013 23706515 10.1016/j.jalz.2013.03.001

[B37] Boccardi M Ganzola R Bocchetta M Pievani M Redolfi A Bartzokis G Camicioli R Csernansky JG de Leon MJ de Toledo-Morrell L Killiany RJ Lehericy S Pantel J Pruessner JC Soininen H Watson C Duchesne S Jack CR Jr Frisoni GB Survey of protocols for the manual segmentation of the hippocampus: preparatory steps towards a joint EADC-ADNI harmonized protocol J Alzheimers Dis 2011 26 61 75 21971451 10.3233/JAD-2011-0004 PMC3829626

[B38] Caroli A Prestia A Chen K Ayutyanont N Landau SM Madison CM Haense C Herholz K Nobili F Reiman EM Jagust WJ Frisoni GB Summary metrics to assess Alzheimer disease-related hypometabolic pattern with 18F-FDG PET: head-to-head comparison J Nucl Med 2012 53 592 600 22343502 10.2967/jnumed.111.094946 PMC3640308

[B39] Nordberg A Carter SF Rinne J Drzezga A Brooks DJ Vandenberghe R Perani D Forsberg A Langstrom B Scheinin N Karrasch M Nagren K Grimmer T Miederer I Edison P Okello A Van Laere K Nelissen N Vandenbulcke M Garibotto V Almkvist O Kalbe E Hinz R Herholz K A European multicentre PET study of fibrillar amyloid in Alzheimer’s disease Eur J Nucl Med Mol Imaging 2013 40 104 114 22961445 10.1007/s00259-012-2237-2 PMC3510420

[B40] Prestia A Caroli A van der Flier WM Ossenkoppele R Van Berckel B Barkhof F Teunissen CE Wall AE Carter SF Scholl M Choo IH Nordberg A Scheltens P Frisoni GB Prediction of dementia in MCI patients based on core diagnostic markers for Alzheimer disease Neurology 2013 80 1048 1056 23390179 10.1212/WNL.0b013e3182872830

[B41] Buerger K Frisoni G Uspenskaya O Ewers M Zetterberg H Geroldi C Binetti G Johannsen P Rossini PM Wahlund LO Vellas B Blennow K Hampel H Validation of Alzheimer’s disease CSF and plasma biological markers: the multicentre reliability study of the pilot European Alzheimer’s Disease Neuroimaging Initiative (E-ADNI) Exp Gerontol 2009 44 579 585 19539742 10.1016/j.exger.2009.06.003

[B42] Kiddle SJ Sattlecker M Proitsi P Simmons A Westman E Bazenet C Nelson SK Williams S Hodges A Johnston C Soininen H Kloszewska I Mecocci P Tsolaki M Vellas B Newhouse S Lovestone S Dobson RJ Candidate blood proteome markers of Alzheimer’s disease onset and progression: a systematic review and replication study J Alzheimers Dis 2014 38 515 531 24121966 10.3233/JAD-130380

[B43] Thambisetty M Simmons A Hye A Campbell J Westman E Zhang Y Wahlund LO Kinsey A Causevic M Killick R Kloszewska I Mecocci P Soininen H Tsolaki M Vellas B Spenger C Lovestone S Plasma biomarkers of brain atrophy in Alzheimer’s disease PLoS One 2011 6 e28527 22205954 10.1371/journal.pone.0028527 PMC3244409

[B44] Furney SJ Kronenberg D Simmons A Guntert A Dobson RJ Proitsi P Wahlund LO Kloszewska I Mecocci P Soininen H Tsolaki M Vellas B Spenger C Lovestone S Combinatorial markers of mild cognitive impairment conversion to Alzheimer’s disease – cytokines and MRI measures together predict disease progression J Alzheimers Dis 2011 26 395 405 21971479 10.3233/JAD-2011-0044

[B45] Simmons A Westman E Muehlboeck S Mecocci P Vellas B Tsolaki M Kloszewska I Wahlund LO Soininen H Lovestone S Evans A Spenger C MRI measures of Alzheimer’s disease and the AddNeuroMed study Ann N Y Acad Sci 2009 1180 47 55 19906260 10.1111/j.1749-6632.2009.05063.x

[B46] Westman E Simmons A Muehlboeck JS Mecocci P Vellas B Tsolaki M Kloszewska I Soininen H Weiner MW Lovestone S Spenger C Wahlund LO AddNeuroMed and ADNI: similar patterns of Alzheimer’s atrophy and automated MRI classification accuracy in Europe and North America Neuroimage 2011 58 818 828 21763442 10.1016/j.neuroimage.2011.06.065

[B47] Westman E Cavallin L Muehlboeck JS Zhang Y Mecocci P Vellas B Tsolaki M Kloszewska I Soininen H Spenger C Lovestone S Simmons A Wahlund LO Sensitivity and specificity of medial temporal lobe visual ratings and multivariate regional MRI classification in Alzheimer’s disease PLoS One 2011 6 e22506 21811624 10.1371/journal.pone.0022506 PMC3141068

[B48] https://aibl.csiro.au/*Australian Imaging, Biomarkers and Lifestyle Flagship Study of Aging* []

[B49] Ellis KA Rowe CC Villemagne VL Martins RN Masters CL Salvado O Szoeke C Ames D Addressing population aging and Alzheimer’s disease through the Australian imaging biomarkers and lifestyle study: collaboration with the Alzheimer’s Disease Neuroimaging Initiative Alzheimers Dement 2010 6 291 296 20451879 10.1016/j.jalz.2010.03.009

[B50] Ellis KA Bush AI Darby D De Fazio D Foster J Hudson P Lautenschlager NT Lenzo N Martins RN Maruff P Masters C Milner A Pike K Rowe C Savage G Szoeke C Taddei K Villemagne V Woodward M Ames D The Australian Imaging, Biomarkers and Lifestyle (AIBL) study of aging: methodology and baseline characteristics of 1112 individuals recruited for a longitudinal study of Alzheimer’s disease Int Psychogeriatr 2009 21 672 687 19470201 10.1017/S1041610209009405

[B51] Rowe CC Ellis KA Rimajova M Bourgeat P Pike KE Jones G Fripp J Tochon-Danguy H Morandeau L O’Keefe G Price R Raniga P Robins P Acosta O Lenzo N Szoeke C Salvado O Head R Martins R Masters CL Ames D Villemagne VL Amyloid imaging results from the Australian Imaging, Biomarkers and Lifestyle (AIBL) study of aging Neurobiol Aging 2010 31 1275 1283 20472326 10.1016/j.neurobiolaging.2010.04.007

[B52] Brown BM Peiffer JJ Sohrabi HR Mondal A Gupta VB Rainey-Smith SR Taddei K Burnham S Ellis KA Szoeke C Masters CL Ames D Rowe CC Martins RN Intense physical activity is associated with cognitive performance in the elderly Transl Psychiatry 2012 2 e191 23168991 10.1038/tp.2012.118 PMC3565765

[B53] Rembach A Faux NG Watt AD Pertile KK Rumble RL Trounson BO Fowler CJ Roberts BR Perez KA Li QX Laws SM Taddei K Rainey-Smith S Robertson JS Vandijck M Vanderstichele H Barnham KJ Ellis KA Szoeke C Macaulay L Rowe CC Villemagne VL Ames D Martins RN Bush AI Masters CL Changes in plasma amyloid beta in a longitudinal study of aging and Alzheimer’s disease Alzheimers Dement 2014 10 53 61 23491263 10.1016/j.jalz.2012.12.006

[B54] Gupta VB Laws SM Villemagne VL Ames D Bush AI Ellis KA Lui JK Masters C Rowe CC Szoeke C Taddei K Martins RN Plasma apolipoprotein E and Alzheimer disease risk: the AIBL study of aging Neurology 2011 76 1091 1098 21422459 10.1212/WNL.0b013e318211c352

[B55] Verdile G Laws SM Henley D Ames D Bush AI Ellis KA Faux NG Gupta VB Li QX Masters CL Pike KE Rowe CC Szoeke C Taddei K Villemagne VL Martins RN Associations between gonadotropins, testosterone and beta amyloid in men at risk of Alzheimer’s disease Mol Psychiatry 2014 19 69 75 23089633 10.1038/mp.2012.147

[B56] Burnham SC Faux NG Wilson W Laws SM Ames D Bedo J Bush AI Doecke JD Ellis KA Head R Jones G Kiiveri H Martins RN Rembach A Rowe CC Salvado O Macaulay SL Masters CL Villemagne VL A blood-based predictor for neocortical Abeta burden in Alzheimer’s disease: results from the AIBL study Mol Psychiatry 2014 19 519 526 23628985 10.1038/mp.2013.40

[B57] Pike KE Ellis KA Villemagne VL Good N Chetelat G Ames D Szoeke C Laws SM Verdile G Martins RN Masters CL Rowe CC Cognition and beta-amyloid in preclinical Alzheimer’s disease: data from the AIBL study Neuropsychologia 2011 49 2384 2390 21529702 10.1016/j.neuropsychologia.2011.04.012

[B58] Lim YY Ellis KA Harrington K Pietrzak RH Gale J Ames D Bush AI Darby D Martins RN Masters CL Rowe CC Savage G Szoeke C Villemagne VL Maruff P Cognitive decline in adults with amnestic mild cognitive impairment and high amyloid-beta: prodromal Alzheimer’s disease? J Alzheimers Dis 2013 33 1167 1176 23160011 10.3233/JAD-121771

[B59] Lim YY Pietrzak RH Ellis KA Jaeger J Harrington K Ashwood T Szoeke C Martins RN Bush AI Masters CL Rowe CC Villemagne VL Ames D Darby D Maruff P Rapid decline in episodic memory in healthy older adults with high amyloid-beta J Alzheimers Dis 2013 33 675 679 23001710 10.3233/JAD-2012-121516

[B60] Villemagne VL Pike KE Chetelat G Ellis KA Mulligan RS Bourgeat P Ackermann U Jones G Szoeke C Salvado O Martins R O’Keefe G Mathis CA Klunk WE Ames D Masters CL Rowe CC Longitudinal assessment of Aβ and cognition in aging and Alzheimer disease Ann Neurol 2011 69 181 192 21280088 10.1002/ana.22248 PMC3045039

[B61] Rowe CC Bourgeat P Ellis KA Brown B Lim YY Mulligan R Jones G Maruff P Woodward M Price R Robins P Tochon-Danguy H O’Keefe G Pike KE Yates P Szoeke C Salvado O Macaulay SL O’Meara T Head R Cobiac L Savage G Martins R Masters CL Ames D Villemagne VL Predicting Alzheimer disease with beta-amyloid imaging: results from the Australian imaging, biomarkers, and lifestyle study of ageing Ann Neurol 2013 74 905 913 24448836 10.1002/ana.24040

[B62] Chetelat G Villemagne VL Villain N Jones G Ellis KA Ames D Martins RN Masters CL Rowe CC Accelerated cortical atrophy in cognitively normal elderly with high beta-amyloid deposition Neurology 2012 78 477 484 22302548 10.1212/WNL.0b013e318246d67a

[B63] Dore V Villemagne VL Bourgeat P Fripp J Acosta O Chetelat G Zhou L Martins R Ellis KA Masters CL Ames D Salvado O Rowe CC Cross-sectional and longitudinal analysis of the relationship between Aβ deposition, cortical thickness, and memory in cognitively unimpaired individuals and in Alzheimer disease JAMA Neurol 2013 70 903 911 23712469 10.1001/jamaneurol.2013.1062

[B64] Chetelat G Villemagne VL Pike KE Ellis KA Bourgeat P Jones G O’Keefe GJ Salvado O Szoeke C Martins RN Ames D Masters CL Rowe CC Independent contribution of temporal β-amyloid deposition to memory decline in the pre-dementia phase of Alzheimer’s disease Brain 2011 134 798 807 21310725 10.1093/brain/awq383

[B65] Chetelat G Villemagne VL Bourgeat P Pike KE Jones G Ames D Ellis KA Szoeke C Martins RN O’Keefe GJ Salvado O Masters CL Rowe CC Relationship between atrophy and beta-amyloid deposition in Alzheimer disease Ann Neurol 2010 67 317 324 20373343 10.1002/ana.21955

[B66] Lim YY Villemagne VL Laws SM Ames D Pietrzak RH Ellis KA Harrington KD Bourgeat P Salvado O Darby D Snyder PJ Bush AI Martins RN Masters CL Rowe CC Nathan PJ Maruff P BDNF Val66Met, Aβ amyloid, and cognitive decline in preclinical Alzheimer’s disease Neurobiol Aging 2013 34 2457 2464 23769397 10.1016/j.neurobiolaging.2013.05.006

[B67] Villain N Chetelat G Grassiot B Bourgeat P Jones G Ellis KA Ames D Martins RN Eustache F Salvado O Masters CL Rowe CC Villemagne VL Regional dynamics of amyloid-beta deposition in healthy elderly, mild cognitive impairment and Alzheimer’s disease: a voxelwise PiB-PET longitudinal study Brain 2012 135 2126 2139 22628162 10.1093/brain/aws125

[B68] Villemagne VL Burnham S Bourgeat P Brown B Ellis KA Salvado O Szoeke C Macaulay SL Martins R Maruff P Ames D Rowe CC Masters CL Amyloid beta deposition, neurodegeneration, and cognitive decline in sporadic Alzheimer’s disease: a prospective cohort study Lancet Neurol 2013 12 357 367 23477989 10.1016/S1474-4422(13)70044-9

[B69] www.j-adni.org/etop.html *Japanese Alzheimer’s Disease Neuroimaging Initiative* []

[B70] Iwatsubo T Japanese Alzheimer’s Disease Neuroimaging Initiative: present status and future Alzheimers Dement 2010 6 297 299 20451880 10.1016/j.jalz.2010.03.011

[B71] Iwatsubo T J-ADNI Rinsho Shinkeigaku 2010 50 935 21921517 10.5692/clinicalneurol.50.935

[B72] Maikusa N Yamashita F Tanaka K Abe O Kawaguchi A Kabasawa H Chiba S Kasahara A Kobayashi N Yuasa T Sato N Matsuda H Iwatsubo T Improved volumetric measurement of brain structure with a distortion correction procedure using an ADNI phantom Med Phys 2013 40 062303 23718605 10.1118/1.4801913

[B73] Yamane T Ikari Y Nishio T Ishii K Kato T Ito K Silverman DH Senda M Asada T Arai H Sugishita M Iwatsubo T Visual–statistical interpretation of 18F-FDG-PET images for characteristic Alzheimer patterns in a multicenter study: inter-rater concordance and relationship to automated quantitative evaluation AJNR Am J Neuroradiol 2014 35 244 249 23907243 10.3174/ajnr.A3665 PMC7965770

[B74] Takahashi R Ishii K Senda M Ito K Kato T Makishi Y Nishio T Ikari Y Iwatsubo T Equal sensitivity of early and late scans after injection of FDG for the detection of Alzheimer pattern: an analysis of 3D PET data from J-ADNI, a multi-center study Ann Nucl Med 2013 27 452 459 23483370 10.1007/s12149-013-0704-x

[B75] Ikari Y Nishio T Makishi Y Miya Y Ito K Koeppe RA Senda M Head motion evaluation and correction for PET scans with 18F-FDG in the Japanese Alzheimer’s Disease Neuroimaging Initiative (J-ADNI) multi-center study Ann Nucl Med 2012 26 535 544 22763629 10.1007/s12149-012-0605-4

[B76] Sevlever G Russo M Vazquez S Gustafson D Surace E Campos J Chrem Mendez P Martin M Martinetto H Ventrice F Guinjoan S Allegri R Argentina ADNI: preliminary report on CSF biomarkers Alzheimers Dement 2013 9 P371 P372

[B77] http://www.alz.org/research/funding/partnerships/ww-adni_meetings.asp#meetings *World Wide Alzheimer’s Disease Neuroimaging Initiative* []

[B78] Lee JH Kim SH Kim GH Seo SW Park HK Oh SJ Kim JS Cheong HK Na DL Identification of pure subcortical vascular dementia using 11C-Pittsburgh compound B Neurology 2011 77 18 25 21593437 10.1212/WNL.0b013e318221acee

[B79] Hsiao IT Huang CC Hsieh CJ Hsu WC Wey SP Yen TC Kung MP Lin KJ Correlation of early-phase (18)F-florbetapir (AV-45/Amyvid) PET images to FDG images: preliminary studies Eur J Nucl Med Mol Imaging 2012 39 613 620 22270508 10.1007/s00259-011-2051-2

[B80] Lin KJ Hsu WC Hsiao IT Wey SP Jin LW Skovronsky D Wai YY Chang HP Lo CW Yao CH Yen TC Kung MP Whole-body biodistribution and brain PET imaging with [18F]AV-45, a novel amyloid imaging agent – a pilot study Nucl Med Biol 2010 37 497 508 20447562 10.1016/j.nucmedbio.2010.02.003

[B81] Yao CH Lin KJ Weng CC Hsiao IT Ting YS Yen TC Jan TR Skovronsky D Kung MP Wey SP GMP-compliant automated synthesis of [(18)F]AV-45 (Florbetapir F 18) for imaging beta-amyloid plaques in human brain Appl Radiat Isot 2010 68 2293 2297 20638295 10.1016/j.apradiso.2010.07.001

[B82] Watt AD Perez KA Rembach AR Masters CL Villemagne VL Barnham KJ Variability in blood-based amyloid-beta assays: the need for consensus on pre-analytical processing J Alzheimers Dis 2012 30 323 336 22426018 10.3233/JAD-2012-120058

[B83] Albert MS Dekosky ST Dickson D Dubois B Feldman HH Fox NC Gamst A Holtzman DM Jagust WJ Petersen RC Snyder PJ Carrillo MC Thies B Phelps CH The diagnosis of mild cognitive impairment due to Alzheimer’s disease: recommendations from the National Institute on Aging-Alzheimer’s Association workgroups on diagnostic guidelines for Alzheimer’s disease Alzheimers Dement 2011 7 270 279 21514249 10.1016/j.jalz.2011.03.008 PMC3312027

[B84] Sperling RA Aisen PS Beckett LA Bennett DA Craft S Fagan AM Iwatsubo T Jack CR Jr Kaye J Montine TJ Park DC Reiman EM Rowe CC Siemers E Stern Y Yaffe K Carrillo MC Thies B Morrison-Bogorad M Wagster MV Phelps CH Toward defining the preclinical stages of Alzheimer’s disease: recommendations from the National Institute on Aging-Alzheimer’s Association workgroups on diagnostic guidelines for Alzheimer’s disease Alzheimers Dement 2011 7 280 292 21514248 10.1016/j.jalz.2011.03.003 PMC3220946

[B85] McKhann GM Knopman DS Chertkow H Hyman BT Jack CR Jr Kawas CH Klunk WE Koroshetz WJ Manly JJ Mayeux R Mohs RC Morris JC Rossor MN Scheltens P Carrillo MC Thies B Weintraub S Phelps CH The diagnosis of dementia due to Alzheimer’s disease: recommendations from the National Institute on Aging-Alzheimer’s Association workgroups on diagnostic guidelines for Alzheimer’s disease Alzheimers Dement 2011 7 263 269 21514250 10.1016/j.jalz.2011.03.005 PMC3312024

[B86] Jack CR Jr Knopman DS Weigand SD Wiste HJ Vemuri P Lowe V Kantarci K Gunter JL Senjem ML Ivnik RJ Roberts RO Rocca WA Boeve BF Petersen RC An operational approach to National Institute on Aging-Alzheimer’s Association criteria for preclinical Alzheimer disease Ann Neurol 2012 71 765 775 22488240 10.1002/ana.22628 PMC3586223

[B87] Jack CR Jr Wiste HJ Lesnick TG Weigand SD Knopman DS Vemuri P Pankratz VS Senjem ML Gunter JL Mielke MM Lowe VJ Boeve BF Petersen RC Brain beta-amyloid load approaches a plateau Neurology 2013 80 890 896 23446680 10.1212/WNL.0b013e3182840bbe PMC3653215

